# Gypensapogenin I alleviates PANoptosis, ferroptosis, and oxidative stress in myocardial ischemic–reperfusion injury by targeting the NOX2/AMPK pathway

**DOI:** 10.3389/fcell.2025.1623846

**Published:** 2025-07-22

**Authors:** Yuqiong Chen, Bo Guan, Jian Lu, Xiaopei Yan, Chao Huang, Yuli Qiu, Xinyan Li, Xiangyu Sun, Lin Chen, Wei Li, Wenjun Mao, Zhongqi Sun, Bin Xu, Su Li, Chao Chen

**Affiliations:** ^1^Department of Cardiology, The Affiliated Suzhou Hospital of Nanjing Medical University, Suzhou Municipal Hospital, Gusu School of Nanjing Medical University, Nanjing, China; ^2^Department of Geriatrics, The Affiliated Suzhou Hospital of Nanjing Medical University, Suzhou Municipal Hospital, Gusu School of Nanjing Medical University, Nanjing, China; ^3^Department of Emergency and Critical Care Medicine, The Affiliated Suzhou Hospital of Nanjing Medical University, Suzhou Municipal Hospital, Gusu School of Nanjing Medical University, Nanjing, China; ^4^Department of Respiratory Medicine, The Affiliated Suzhou Hospital of Nanjing Medical University, Suzhou Municipal Hospital, Gusu School of Nanjing Medical University, Nanjing, China; ^5^Ministry of Science and Technology, The Affiliated Suzhou Hospital of Nanjing Medical University, Suzhou Municipal Hospital, Suzhou, Jiangsu, China; ^6^Department of Nephrology, The Affiliated Suzhou Hospital of Nanjing Medical University, Suzhou Municipal Hospital, Suzhou, China; ^7^National Cancer Center/National Clinical Research Center for Cancer/Cancer Hospital, Chinese Academy of Medical Sciences and Peking Union Medical College, Beijing, China; ^8^Department of Cardiology, Suzhou WuZhong People’s Hospital, Suzhou, Jiangsu, China; ^9^Department of Cardiology, Shanghai Institute of Cardiovascular Diseases, Zhongshan Hospital, Fudan University, Shanghai, China; ^10^Department of Cardiology, The First Affiliated Hospital of Soochow University, Suzhou, Jiangsu, China

**Keywords:** gypensapogenin I, myocardial ischemia–reperfusion injury, PANoptosis, ferroptosis, oxidative stress, NADPH oxidase 2

## Abstract

**Aim:**

This study aims to investigate the benefits of gypensapogenin I (GI) on myocardial ischemia–reperfusion injury (MIRI) and the underlying mechanisms.

**Methods:**

An MIRI model was established by ligating the anterior descending coronary artery (LAD) followed by blood flow restoration in mice. Cardiac dysfunction and myocardial infarction size were evaluated by echocardiography and triphenyltetrazolium chloride (TTC) staining. PANoptosis, ferroptosis, and mitochondrial redox state were examined by immunofluorescence, Western blotting, and an ELISA kit. In addition, molecular and biochemical methods were applied to illustrate the exact mechanisms of GI on MIRI.

**Results:**

GI pretreatment alleviated cellular oxidative stress, inhibited PANoptosis and ferroptosis, reduced myocardial infarction area, and improved cardiac function during MIRI. Further results revealed that mitochondrial biogenesis and the anti-oxidative system were impaired in mice suffering from MIRI, and these effects were significantly alleviated by GI treatment via downregulation of the NADPH oxidase 2 (NOX2) level. Moreover, NOX2 promoted mitochondrial dysfunction by suppressing the AMP-activated protein kinase (AMPK)–PGC-1α–Sirt3 signaling pathway. In addition, the NOX2 activator exacerbated oxidative damage and offset all the beneficial effects of GI on mitochondrial function, PANoptosis, and ferroptosis. Meanwhile, reinforced AMPK phosphorylation by GI or AMPK activator (5-aminoimidazole-4-carboxamide ribonucleotide, AICAR) maintained the mitochondrial redox state and biogenesis and suppressed PANoptosis and ferroptosis.

**Conclusion:**

GI pretreatment protected the cardiomyocytes from MIRI-induced PANoptosis and ferroptosis by maintaining the mitochondrial redox state and biogenesis through the modulation of the NOX2/AMPK signaling pathway. Our findings indicate that GI pretreatment could be a promising therapeutic agent for MIRI treatment.

## Graphical Abstract

**Figure:**
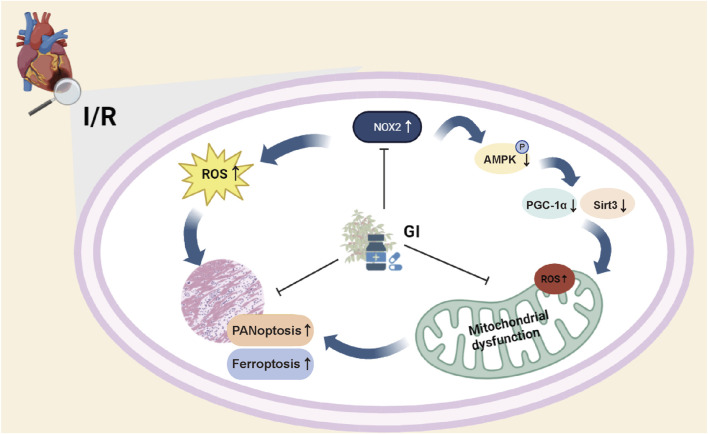
Gypensapogenin I alleviates PANoptosis, ferroptosis, and oxidative stress in myocardial ischemic–reperfusion injury. GI pretreatment attenuated PANoptosis and ferroptosis in cardiomyocytes during MIRI by mitigating NADPH oxidase 2 (NOX2)-dependent oxidative stress and restoring AMPK–PGC-1α–Sirt3 signaling pathway-dependent mitochondrial biogenesis.

## 1 Introduction

Ischemic heart disease (IHD) can progress to acute coronary syndrome (ACS) and ultimately lead to heart failure (HF), both of which impose a severe global health burden (Antman and Braunwald, 2020). It is projected that IHD will become the second leading cause of mortality worldwide by 2030 (Stone et al., 2023). Restoring coronary blood flow is recognized as the standard treatment for myocardial infarction and is now routinely performed in clinical practice (Jamal et al., 2023). However, abrupt reperfusion can paradoxically cause secondary myocardial injury, which is known as myocardial ischemia–reperfusion injury (MIRI) (Liu M. et al., 2024; Hausenloy and Yellon, 2013). MIRI not only expands the infarction area but also increases perioperative mortality, significantly counteracting the benefits of reperfusion therapy (Hausenloy and Yellon, 2013; Forman et al., 1989; Li et al., 2021). Massive cardiomyocyte programmed cell death and mitochondrial dysfunction are the hallmark pathological features of MIRI (Xiang et al., 2024a). However, the precise underlying mechanisms remain incompletely understood, underscoring the critical need for further research into myocardial protection strategies against MIRI.

Multiple cell death modalities, including pyroptosis, apoptosis, ferroptosis, and necroptosis, participated in cardiac ischemia–reperfusion injury, with intricately interconnected pathogenic pathways (Li et al., 2021; Cai et al., 2023; Liu S. et al., 2024; Zh et al., 2024). Malireddi et al. (2019) proposed the concept of PANoptosis, highlighting the concurrent activation and mechanistic interplay of pyroptosis, apoptosis, and necroptosis in inflammatory diseases. As a newly identified lytic cell-death pathway, PANoptosis is mediated by a multiprotein complex that activates pyroptosis caspases (e.g., caspase-1), apoptotic caspases (e.g., caspase-8), and receptor-interacting serine-threonine protein kinases 3 (RIPK3)/mixed lineage kinase domain like (MLKL)-driven necroptosis. Unlike isolated forms of cell death, PANoptosis is triggered by specific stimuli (e.g., ischemia and cytokine storms) and amplifies inflammation through the release of damage associated molecular patterns (DAMPs) and cytokines (IL-1β and IL-18) (Nadella and Kanneganti, 2024). Previous studies have demonstrated that a combinatorial inhibition of multiple cell-death pathways (e.g., pyroptosis, apoptosis, and necroptosis) significantly enhances cell survival compared to single-pathway inhibition (Yan et al., 2023). Therefore, targeting PANoptosis in MIRI may maximize therapeutic benefits by simultaneously inhibiting multiple cell-death pathways. However, current investigations targeting PANoptosis in ischemia–reperfusion were largely confined to neurology (She et al., 2023). Given the established benefits of inhibiting PANoptosis in doxorubicin-induced cardiotoxicity, targeting PANoptosis may represent a promising strategy for alleviating MIRI (Bi et al., 2022a; Li et al., 2024; Ge et al., 2025; Gao et al., 2024).

The hallmark feature of PANoptosis is the assembly of the PANoptosome—a molecular scaffold that integrates the core components of multiple cell-death pathways and is critical for driving subsequent cell death events (Sundaram et al., 2024). However, the exact machinery regulating the PANoptosome remains unclear, and substantial evidence confirms mitochondrial dysfunction as a key regulator of multiple cell-death pathways (She et al., 2023). Currently, mitochondrial-targeted therapies have shown promising efficacy in mitigating programmed cell death and MIRI (Marin et al., 2021; Li et al., 2023; Chen et al., 2021). However, whether mitochondria represent a promising target for PANoptosome/PANoptosis inhibition during MIRI remains to be elucidated.

Jiaogulan (*Gynostemma pentaphyllum* (Thunb.) Makino) is a widely distributed herbal plant in Asia that has been traditionally used as a folk medicine since the Ming Dynasty (Xie et al., 2012; Lee et al., 2025). Recently, several studies have also presented the salutary effects of Jiaogulan on preventing multiple diseases (Huang et al., 2019; Huang et al., 2022). Further pharmacological investigations revealed that the bioactive components of Jiaogulan possess anti-inflammatory, anti-oxidative, and anti-arteriosclerotic properties (Cai et al., 2016; Yu et al., 2016; Gou et al., 2016). A phytochemical investigation of G. pentaphyllum yielded four secondary hydrolysates, designated as gypensapogenins (Zhang et al., 2019). All gypensapogenins significantly enhanced H9c2 cardiomyocyte survival under H_2_O_2_-induced apoptosis, outperforming even vitamin E (Zhang et al., 2019). In addition, gypensapogenin I (GI) was reported to improve isoproterenol (ISO)-induced cardiac tissue inflammation and myocardial death (Li et al., 2022). Therefore, it is important to investigate whether GI could also confer protective effects against MIRI.

In this study, we investigated the therapeutic potential of GI in mitigating MIRI and the associated cardiomyocyte death. Our results demonstrated that GI confers cardioprotection by simultaneously inhibiting PANoptosis and ferroptosis while attenuating mitochondrial oxidative stress through NADPH oxidase 2 (NOX2) suppression. These findings established GI as a promising therapeutic strategy against MIRI.

## 2 Methods

### 2.1 Reagents and antibodies

The bioactive component extracted form Jiaogulan was purchased from Hunan Chunguang Jiuhui Modern Chinese Medicine Co., Ltd. The raw material was further subjected to hydrolysis and chromatography, which were performed by YiSheng Pharmaceutical Co., Ltd (Ji’an, Jilin Province, China). Finally, GI, prepared as a white amorphous solid with a purity of up to 98.5%, was used in the following experiments.

GKT137839 and Compound C were used as NOX2 and AMPK inhibitors, respectively, and were purchased from MCE (United States). Tetrabromocinnamic acid (TBCA) was used as the NOX2 activator and was obtained from MCE. The compound 5-aminoimidazole-4-carboxamide ribonucleotide (AICAR) was used as the AMPK activator and was purchased from MCE. The lactate dehydrogenase (LDH) kit, creatine kinase MB isoenzyme (CK-MB) kit, troponin (cTnI) kit, myoglobin (Myo) kit, and alanine aminotransferase (ALT) kit were ordered from Jiancheng Bioengineering Institute (Nanjing, China). Antibodies against caspase-3, cleaved-caspase-3, RIP1, RIP3, p-RIP3, MLKL, p-MLKL, and GAPDH were obtained from Abcam (Cambridge, United Kingdom). Anti-AMPK, anti-PGC-1α, and anti-Sirt3 were obtained from Cell Signaling Technologies (Danvers, United States). Other primary antibodies and secondary antibodies were purchased from Proteintech (Shanghai, China), unless otherwise specified.

### 2.2 Animals and therapeutical treatment

All experimental protocols were reviewed and sanctioned by the Animal Experimental Ethics Committee, and all procedures were strictly performed in accordance with guidelines of Nanjing Medical University to minimize unnecessary animal suffering. Eight-week-old male C57BL/6 mice (weighing 20–24 g) were purchased from M.Q. MICROBE Co., Ltd (Suzhou, China). They were acclimatized to a temperature-controlled (25°C ± 5°C) cage with a 12 h light/dark cycle and 50% relative humidity for at least 1 week. During the whole time, the mice were given *ad libitum* access to water and food. For the GI pretreatment study, mice were randomly allocated into groups (n = 12 per group) using a simple randomization method (Del Castillo et al., 2021): sham, sham + GI, I/R, and I/R + GI. GI was administered intraperitoneally (ip) at 10, 20, and 30 mg/kg once daily for 3 weeks before MIRI surgery (Li et al., 2022; Tan et al., 2022). For the NOX2 rescue experiment, mice were randomly divided into five groups (n = 12 per group): sham, IR, IR + GI, IR + GSK2795039 (100 mg/kg, ip) (Hirano et al., 2015), and IR + GI + TBCA (10 mg/kg, ip) (Wang et al., 2018).

### 2.3 Surgical procedures

The MIRI mouse model was established by temporary constriction and blood flow restoration of the left anterior descending (LAD) coronary artery, according to Gao et al. (2010). In brief, after thoracic hair removal, the mice were anesthetized by 100% O_2_/4% isoflurane inhalation and then put on a 37°C homothermal pad. After sterilization, the mice were masked and maintained under sedation with 100% O_2_/2% isoflurane throughout the whole operation. A thoracic incision was made on the fourth intercostal space, and then the heart was exposed by compression of the thoracic cavity. The LAD was constricted by a 7–0 silk suture slipknot for 30 min, after which the slipknot was loosened to restore blood flow. The sham group was only subjected to threading without any narrowing of the coronary artery. All surgical procedures and evaluations were performed by investigators blinded to treatment-group assignments.

### 2.4 Echocardiography

After 24 h of reperfusion, the mice were subjected to ultrasound before being euthanized. A Vevo 2100 High-Resolution Imaging System equipped with a 30 MHz sensor was used for capturing the image on the parasternal left ventricular long- and short-axis view, from which the left ventricular internal diastole/systole dimension (LVIDd/LVISd) was measured, and the left ventricular fractional shortening (LVFS) and left ventricular ejection fraction (LVEF) were correspondingly calculated.

### 2.5 Triphenyltetrazolium chloride staining

After anesthesia with 2% isoflurane, mouse hearts were rapidly harvested, sectioned into 1-mm slices, and incubated in 1% triphenyltetrazolium chloride (TTC) solution (Sigma, United States) at 37°C for 10 min. Consecutive slices were scanned using a white light scanner (Canon, Japan). Infarcted myocardium appeared white, while viable myocardium was red. The infarction degree was calculated as the ratio of the infarcted area to the left ventricular free wall area.

### 2.6 Measurement of oxidative damage biomarkers

Protein samples were extracted from the border zone of cardiac tissues, cardiomyocytes, or isolated mitochondria via lysis and centrifugation and quantified using a BCA Protein Assay Kit (Beyotime, China). Malondialdehyde (MDA) content was measured using a malondialdehyde assay kit (Nanjing Jiancheng Bio, China). Glutathione (GSH) and oxidized glutathione (GSSG, glutathione disulfide) concentrations were determined using GSH and GSSG assay kits (Beyotime, China). The enzyme activities of catalase (CAT) and thioredoxin reductase (TRXR) were quantified using CAT and TRXR test kits (Nanjing Jiancheng Bio, China). Sulfhydryl (SH) content was measured using an SH content detection kit, and non-protein SH (NPSH) concentration was determined following the Sedlak and Lindsay method (1968) (Sanchez-Moya et al., 2022). Superoxide dismutase (SOD) and manganese-SOD (Mn-SOD) activities were measured using Cu/Zn-SOD and Mn-SOD assay kits with the WST-8 method (Beyotime, China).

### 2.7 Fluorescence staining

Primary cardiomyocytes were cultured in confocal dishes. Mitochondria were stained with 100 nM MitoTracker Red CMXRos (Beyotime) for 30 min at 37°C in the dark. Propidium iodide (PI) staining and ethidium homodimer III (EthD-III; Biotium, United States) staining were performed according to previously published protocols (Yan et al., 2023; Chen Z. et al., 2024). Cells were stained with 5 mg/mL Hoechst 33342 and 10 mg/mL PI for 10 min in the dark. EthD-III was dissolved in 1 × PBS at 1 μg/mL. Cells were treated with EthD-III solution for 10 min at room temperature and fixed with 4% paraformaldehyde for 20 min. TUNEL staining on paraffin sections and cells was performed using the TUNEL Apoptosis Assay Kit (Beyotime Biotechnology, Beijing, China) according to the product instructions. Samples were observed and imaged under a confocal microscope, and the positive cells were counted using ImageJ software (version 1.53c, NIH).

### 2.8 Immunofluorescence staining

To determine the oxidative stress of the cardiac tissue, SOD and acylated superoxide dismutase (Ac-SOD) levels were measured through immunofluorescence. The cardiac tissue sections were first blocked with 5% BSA and then subjected to SOD and Ac-SOD antibody overnight. After incubating with fluorescent secondary antibodies and DAPI dyeing on the second day, a fluorescence microscope was used for imaging, and the mean intensity quantitative analyses were carried out via ImageJ software (version 1.53c, NIH, United States).

### 2.9 Molecular docking assay

To evaluate the molecular binding potential of GI with NOX2, the 3D structure of GI was obtained from PubChem (https://pubchem.ncbi.nlm.nih.gov/) (Kim et al., 2023). The protein structure of NOX2 was predicted using AlphaFold 3 (Jumper et al., 2021). Molecular docking was performed with Schrödinger Maestro (v2022-1) using the Glide module with the default parameters, unless otherwise specified. Docking poses were evaluated based on docking scores (GlideScore) and binding energy (MM/GBSA). A docking score threshold of ≤ −5.0 kcal/mol was set to identify high-affinity interactions. Protein–ligand interactions were analyzed using Protein–Ligand Interaction Profiler (PLIP; https://plip-tool.biotec.tu-dresden.de) and visualized in PyMOL (https://www.pymol.org/, v2.5).

### 2.10 Cellular thermal shift assay

H9C2 cells were collected and freeze-thawed under liquid nitrogen, and cell lysates were diluted with PBS and divided into two aliquots, with one aliquot being added to GI and the other being used as a DMSO control. After incubation at 37°C for 1 h, all lysates were equally divided into nine PCR tubes, and each tube of cells was heated separately at different temperatures (37°C, 40°C, 43°C, 49°C, 52°C, 55°C, 58°C, 61°C, and 64°C) for 3 min. After that, samples were centrifuged at 4°C, and the soluble fractions were subjected to Western blot analysis (Fu et al., 2025; Ma et al., 2022).

### 2.11 Cell isolation and viability assay

Primary neonatal mouse ventricular cardiomyocytes (NMVCs) were extracted according to Shi et al. (2021). In brief, neonatal mice within 1 day were purchased from the M.Q. MICROBE Co., Ltd. (Suzhou, China). After sterilization, a horizontal incision was made at the level of the rib, and the heart was pumped out by compression of the chest cavity. The heart was then minced and enzymatically dissociated using Liberase (Roche, Basel, Switzerland). The cell suspension was filtered through a 70-µm nylon strainer (BD Falcon, Franklin Lakes, NJ) and incubated in DMEM supplemented with 10% fetal bovine serum (FBS) for 1 h at 37°C/5% CO_2_ to facilitate differential plating. Cardiac fibroblasts adhered to the culture flask, while NMVCs remained in suspension. NMVCs were then separated and cultured in high-glucose DMEM containing 10% FBS and 1% penicillin–streptomycin.

The cellular hypoxia/reoxygenation (H/R) injury model was established by oxygen and glucose deprivation, as reported in previous studies (Chen et al., 2025). NMVCs were incubated with ischemic medium and subjected to hypoxia in a sealed chamber (Billups–Rothenberg, San Diego, United States) maintained at 1% O_2_ (balanced with 94% N_2_ and 5% CO_2_) for 12 h. To simulate reoxygenation injury, cells were then reoxygenated by replacing the ischemic medium with fresh normoxic culture medium and incubating under standard conditions (21% O_2_ and 5% CO_2_) for 6 h.

Cell viability was tested using a CCK-8 Cell Counting Kit obtained from Biosharp (Shanghai, China). In brief, CCK-8 working solution (10 μL) was added into the culture medium of each required well at 37°C for 2–4 h. Then, the absorption value at 450 nm of each sample was quantified and analyzed using a microplate reader (Thermo Fisher Scientific, United States).

### 2.12 Western blotting

Total proteins from cardiac tissue and cells were extracted using RIPA lysis buffer; after measuring protein concentration using a BCA Protein Assay Kit, the samples were loaded onto a precast 8%–12% SDS-PAGE gel. After electrophoresis, separated proteins were transferred to an activated PVDF membrane and blocked with skim milk. After sealing, the PVDF membrane was incubated overnight with different primary antibodies according to the study design. Finally, after incubation with secondary antibodies, the blots were detected using the ECL chemiluminescent system (Seyotin, China) . Detailed information on the antibodies used for Western blotting is presented in Supplementary Table S1.

### 2.13 Real-time quantitative PCR

RNA in each sample was extracted following the instructions of the Total RNA Extraction Kit (Solarbio, China), and then the mRNAs were transcribed to complementary DNA (cDNA) using a cDNA reverse transcription kit (Invitrogen, United States). Real-time quantitative PCR (RT-qPCR) was performed using the CFX96 Real-Time PCR System and SYBR Green I (TSE202, TSINGKE). The 2^−ΔΔCT^ method was used to calculate the relative expression of the required genes.

### 2.14 Statistical analysis

The data derived from biological replicates were presented as the mean ± SEM. The data were analyzed using GraphPad Prism software (version 10.0, GraphPad Software, United States). Normality was assessed using the Shapiro–Wilk and Kolmogorov–Smirnov tests. Student’s t-test was applied when comparing means from two groups, while one-way ANOVA was applied for multiple comparisons. A *p*-value <0.05 was considered statistically significant.

## 3 Results

### 3.1 GI alleviated cardiac injury and cell death during MIRI in mice

**FIGURE 1:**
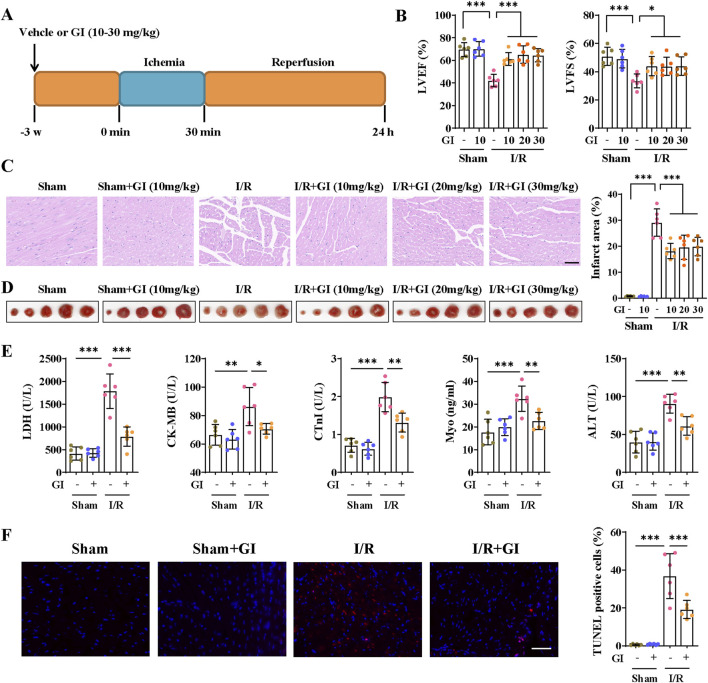
GI alleviated cardiac injury and cell death during MIRI. **(A)** Time schedule of GI pretreatment and MIRI procedure. **(B)** Echocardiographic study was performed 24 h after MIRI, and the LVEF and LVFS were statistically analyzed. **(C)** Representative images of HE staining. Scale bars = 40 μm. **(D)** Representative images of TTC staining and quantitative analysis of infarction size. **(E)** Effects of GI on serum levels of LDH, CK-MB, cTnI, Myo, and ALT. **(F)** Representative images and quantitative analysis of TUNEL staining of cardiac samples. Scale bars = 40 μm. Six biological replicates were included in the experiment. Data are expressed as the mean ± standard error (SEM). One-way ANOVA test was applied for multi-group comparisons. * (p < 0.05), ** (p < 0.01), and *** (p < 0.001). Gypensapogenin I (GI), left ventricular ejection fraction (LVEF), left ventricular fractional shortening (LVFS), lactate dehydrogenase (LDH), creatine kinase MB isoenzyme (CK-MB), troponin (cTnI), myoglobin (Myo), alanine aminotransferase (ALT), and creatine (Cr).

Mice were subjected to the pretreatment of vehicle or GI (10, 20, and 30 mg/kg) 3 weeks prior to MIRI, and MIRI was induced by 30-min ligation of the LAD followed by 24 h of reperfusion (Figure 1A) (Li et al., 2022). Each mouse underwent M-mode cardiography before being euthanized. MIRI mice showed lower LVFS and LVEF than sham mice, which indicated impaired cardiac function (Figure 1B). GI pretreatment significantly alleviated cardiac dysfunction during MIRI (Figure 1B). Moreover, H/E staining showed that mice suffering from MIRI exhibited cardiac disorganization, and TTC staining also showed an infarction area (Figures 1C, D). In contrast, GI pretreatment alleviated cardiac disorganization and reduced the infarction area (Figures 1C, D). We also confirmed that 10 mg/kg/day of GI is the most effective concentration, with no additional benefits observed at higher doses; therefore, 10 mg/kg/day of GI was selected for subsequent *in vivo* experiments. Elevated serum levels of LDH, CK-MB, cTnI, Myo, and ALT, along with TUNEL-positive cells, were ameliorated by 10 mg/kg/day GI treatment (Figures 1E, F). Collectively, these results indicated that GI exerted a cardiac protective role against MIRI.

### 3.2 GI ameliorated mitochondrial oxidative stress and conserved mitochondrial biogenesis in mice suffering from MIRI

**FIGURE 2:**
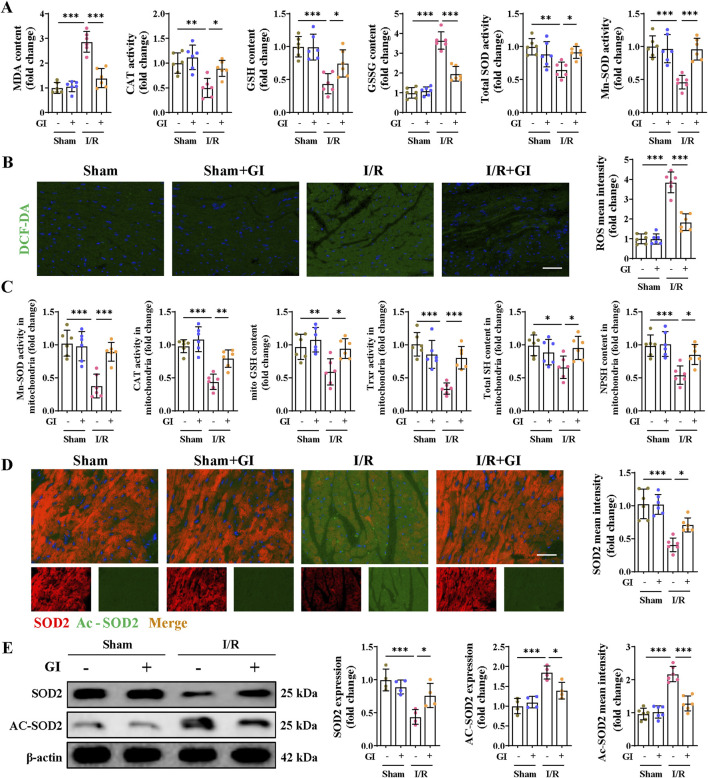
Effects of GI on MIRI-induced oxidative stress. **(A)** Contents of MDA, GSH, and GSSG and the enzyme activities of CAT and SOD in heart tissues. **(B)** Representative ROS fluorescence images and mean intensities of the cardiac tissues. **(C)** Contents of GSH, SH, and NPSH and the enzyme activities of Mn-SOD and TRXR in mitochondria isolated from heart tissues. **(D)** Immunofluorescence staining and mean intensity of SOD2 and Ac-SOD2 in cardiac tissues. **(E)** Western blot analysis detected the expression of SOD2 and Ac-SOD2. Four to six biological replicates were included in the experiment. Data are expressed as the mean ± standard error (SEM). One-way ANOVA test was applied for multi-group comparisons. * (p < 0.05), ** (p < 0.01), and *** (p < 0.001). Malondialdehyde (MDA), catalase (CAT), glutathione (GSH), glutathione disulfide (GSSG), superoxide dismutase (SOD), acetylated superoxide dismutase 2 (Ac-SOD2), manganese superoxide dismutase (Mn-SOD), and thioredoxin reductase (TRXR).

Excessive oxidative damage plays a vital role in the emergence and development of MIRI; we then examined whether GI treatment (10 mg/kg/day) can improve reperfusion injury-induced oxidative stress. GSH, CAT, and SOD are three essential oxidoreductases reflecting anti-oxidative capacity. GSSG is the oxidized form of GSH, and MDA is a lipid peroxidation product, both of which can reflect cellular oxidative burden. Mice in the MIRI group presented significantly higher levels of reactive oxygen species (ROS), MDA, and GSSG and depressed levels of GSH, CAT, and SOD. However, all the oxidative stress injuries were remarkably reversed by GI treatment (Figures 2. A, B).

Mn-SOD, a mitochondrial matrix isoform of SODs*,* exerts superoxide scavenging properties in the mitochondrial matrix*.* Its decrease in the MIRI group suggests severe mitochondrial oxidative stress during MIRI*.* We, therefore, examined the antioxidant system capacities in mitochondria and found that Mn-SOD, CAT, and TRXR all showed mitigated activities in the MIRI group. A similar trend was also observed in the content of GSH, total thiol (SH), and non-protein thiol levels (NPSH). However, GI treatment remarkably salvaged the capacity of the above enzymes in MIRI mice (Figure 2C). In addition, downregulated expression of SOD2 and elevated expression of Ac-SOD2 were observed in the MIRI group, which were also reversed by GI pretreatment (Figures 2D, E). These findings support the potential of GI to reduce mitochondrial oxidative damage in MIRI.

**FIGURE 3:**
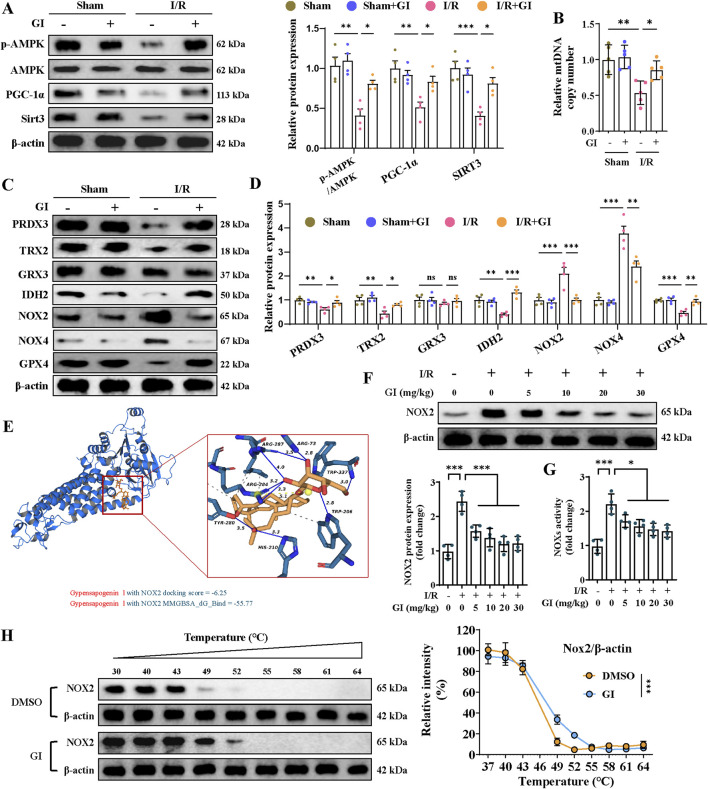
Effects of GI on mitochondrial biogenesis and antioxidant system in MIRI. **(A)** Western blots of p-AMPK, PGC-1α, and Sirt3 in the cardiac tissue. **(B)** Quantification of relative mtDNA copy number in the cardiac tissue. **(C, D)** Representative blots and quantitative analysis of mitochondrial antioxidant/pro-oxidant-related proteins. **(E)** Molecular docking between NOX2 and GI. **(F)** Western blots of NOX2 expression under treatment of GI (0 mg/kg, 5 mg/kg, and 10 mg/kg). **(G)** Statistic analysis of NOX activity. **(H)** Cellular thermal shift assay detecting the thermal stability of NOX2 after GI treatment in H9C2. Four to six biological replicates were included in the experiment. Data are expressed as the mean ± standard error (SEM). One-way ANOVA test was applied for multi-group comparisons. * (p < 0.05), ** (p < 0.01), and *** (p < 0.001). Phosphorylated AMP-activated protein kinase (p-AMPK), peroxisome proliferator-activated receptor gamma coactivator-1 alpha (PGC-1α), and NADPH oxidase 2 (NOX2).

Previous studies show that oxidative stress-induced mitochondrial damage is closely linked to mitochondrial biogenesis, which is primarily regulated by the AMPK/PGC-1α/Sirt3 pathway (Wang et al., 2022; Wu et al., 2021). The present work showed that the p-AMPK/AMPK ratio and the levels of PGC-1α and Sirt3 in the MIRI group were remarkably downregulated, while supplementation with GI preserved the phosphorylation level of AMPK and the expression of PGC-1α and Sirt3 (Figure 3A). Similarly, MIRI reduced the mtDNA copy number, which was restored by GI pretreatment (Figure 3B). Together, our results suggest that GI ameliorates mitochondrial oxidative damage during MIRI by preserving mitochondrial biogenesis via the AMPK–PGC-1α–Sirt3 pathway.

### 3.3 GI protected against cellular redox stress during MIRI by interacting with NOX2

Further studies were conducted to elucidate the molecular mechanisms underlying GI’s protective effects. The MIRI group exhibited significant oxidative stress, characterized by elevated NOX2 and NOX4 protein levels, along with reduced expression of peroxiredoxin 3 (PRDX3), thioredoxin 2 (TRX2), isocitrate dehydrogenase 2 (IDH2), and glutathione peroxidase 4 (GPX4). Notably, GI pretreatment effectively reversed these alterations (Figures Fig 3C, D). Molecular docking analysis identified NOX2 as a potential binding target of GI (Figure 3E). We found that the protein level of NOX2 and NOX activity decreased in a dose-dependent manner with GI pretreatment (Figures 3F, G). Furthermore, the biological interaction of NOX2 with GI was demonstrated using the cellular thermal shift assay (CETSA), which showed that GI incubation led to the stabilization of NOX2 (Figure 3H).

**FIGURE 4:**
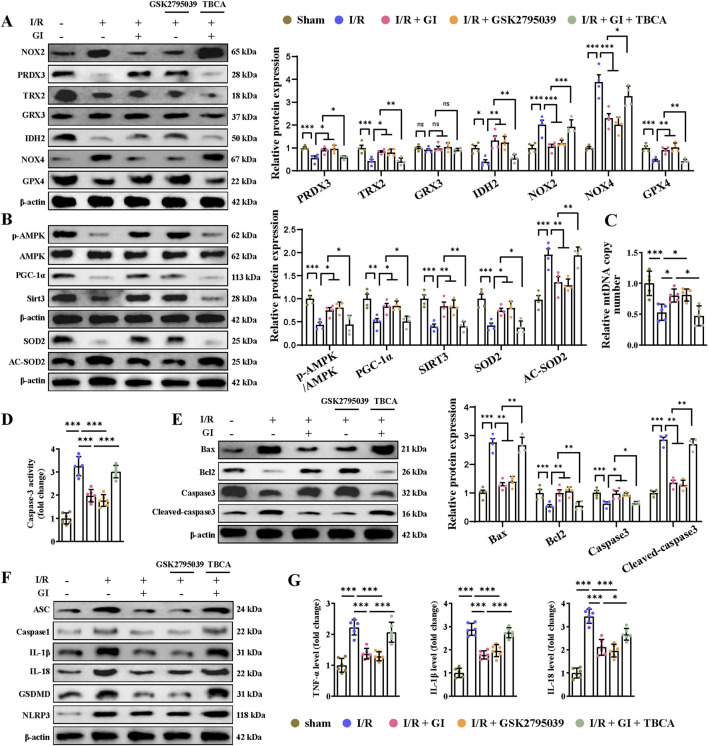
Activation of NOX2 abolished the protective effects of GI on MIRI-induced mitochondrial dysfunction, apoptosis, and pyroptosis. **(A)** Western blots presenting mitochondrial antioxidant/pro-oxidant-related proteins. **(B)** Western blots of p-AMPK, PGC-1α, and Sirt3 in the cardiac tissue. **(C)** Quantification of relative mtDNA copy number in the cardiac tissue. **(D)** Statistical analysis of caspase-3 activity. **(E)** Western blotting of caspase-3, cleaved-caspase-3, Bax, and Bcl-2 in cardiac tissues. **(F)** Western blot analysis of NLRP3, ASC, caspase-1, and GSDMD, IL-1β, and IL-18. **(G)** ELISA analysis of serum levels of TNF-α, IL-1β, and IL-18. Four to six biological replicates were included in the experiment. Data are expressed as the mean ± standard error (SEM). One-way ANOVA test was applied for multi-group comparisons. * (p < 0.05), ** (p < 0.01), and *** (p < 0.001). NLR family pyrin domain containing 3 (NLRP3) and gasdermin D (GSDMD).

To determine whether NOX2 mediated GI’s protective effects against MIRI, we then applied the NOX2 activator (TBCA) and inhibitor (GSK2795039). We found that by suppressing the level of NOX2 with GSK2795039, NOX4 expression was reduced accordingly, while other anti-oxidative proteins, including PRDX3, TRX2, IDH2, and GPX4, were increased (Figure 4A). In contrast, the NOX2 activator TBCA abolished all the benefits of GI on the regulation of oxidative stress-related proteins (Figure 4A). The results indicated that NOX2 is potentially the direct target of GI.

We then examined the involvement of NOX2 in GI-exerted mitochondrial protection and oxidative stress inhibition. NOX2 activator TBCA could significantly offset the anti-oxidative effect of GI by suppressing the expression of SOD2 and elevating the expression of Ac-SOD2 (Figure 4B). Conversely, the NOX2 inhibitor GSK2795039 showed similar benefits to GI in inhibiting oxidative stress (Figure 4B). Furthermore, GSK2795039 enhanced AMPK–PGC-1α–Sirt3 signaling and increased the mtDNA levels (Figures 4B, C). In contrast, TBCA abolished GI’s protective effects on mitochondrial biogenesis (Figures 4B, C). Together, these results identify NOX2 as a key regulator of GI-mediated redox balance and mitochondrial protection during MIRI.

### 3.4 GI mitigated programmed cell death during MIRI by downregulating NOX2

**FIGURE 5:**
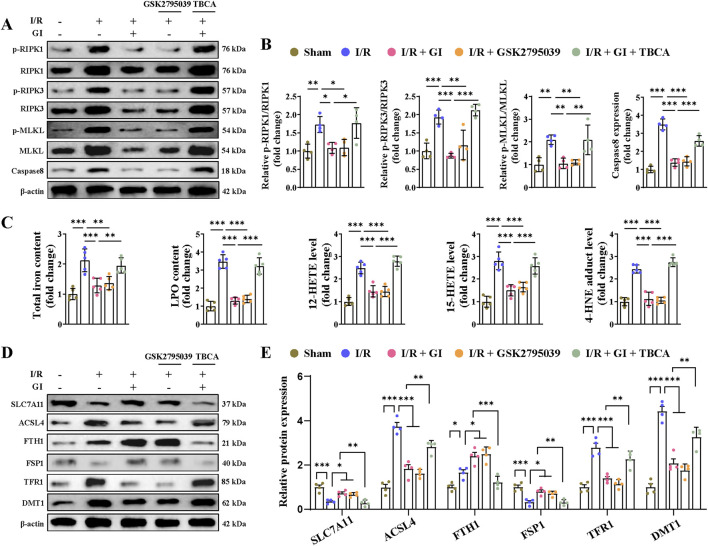
Activation of NOX2 abolished the protective effects of GI on MIRI-induced necroptosis and ferroptosis. **(A, B)** Western blot analyses of relative p-RIPK1, p-RIPK3, p-MLKL, and caspase levels in cardiac tissues. **(C)** Contents of total iron and LPO and the levels of 12-HETE, 15-HETE, and 4-HNE in heart tissues. **(D, E)** Western blotting of ferroptosis-related proteins. Four to six biological replicates were included in the experiment. Data are expressed as the mean ± standard error (SEM). One-way ANOVA test was applied for multi-group comparisons. * (p < 0.05), ** (p < 0.01), and *** (p < 0.001). Phosphorylated receptor-interacting serine-threonine protein kinases 1 (p-RIPK1), phosphorylated receptor-interacting serine-threonine protein kinases 3 (p-RIPK3), phosphorylated mixed lineage kinase domain like (p-MLKL), lipid peroxidation (LPO), and 4-hydroxynonenal (4-HNE).

Multiple forms of programmed cell death contribute to MIRI (Xiang et al., 2024a). Recently, PANoptosis, a novel and interconnected cell-death pathway involving pyroptosis, apoptosis, and necroptosis, has been implicated in cardiac injury and cardiomyocyte death (Bi et al., 2022b; Xiang et al., 2024b). Ferroptosis has also been reported in MI and MIRI (Liu et al., 2023; Yu et al., 2023). However, it remains unclear whether NOX2 participates in regulating these cell death pathways and whether GI protects against MIRI by inhibiting PANoptosis and ferroptosis. Our results demonstrated that MIRI induced apoptosis, as shown by increased caspase-3 activity, elevated cleaved-caspase-3 and Bax levels, and reduced Bcl-2 expression (Figures 4D, E). GI pretreatment reversed all these apoptotic injuries (Figures 4D, E). The NOX2 inhibitor GSK2795039 produced similar protective effects as GI, while the NOX2 activator TBCA abolished GI’s anti-apoptotic effects (Figures 4D, E). Pyroptosis was also activated during MIRI, which was evidenced by elevated serum and tissue levels of TNF-α, IL-1β, and IL-18, along with the increased expression of caspase-1, ASC, NLR family pyrin domain containing 3 (NLRP3), and gasdermin D (GSDMD) in cardiac tissue (Figures 4F, G). GI pretreatment suppressed pyroptosis by normalizing these changes (Figures 4F, G). Notably, NOX2 inhibition with GSK2795039 replicated GI’s effects, while NOX2 activation with TBCA blocked GI’s protection (Figures 4F, G). Furthermore, we observed that GI and GSK2795039 pretreatment significantly attenuated MIRI-induced necroptosis by reducing the phosphorylation of receptor-interacting serine-threonine protein kinases 1 (RIPK1), RIPK3, and MLKL and suppressing caspase-8 expression (Figures 5A, B). In contrast, TBCA co-treatment completely abolished GI’s protective effects against necroptosis (Figures 5A, B).

As previously demonstrated, GI significantly elevated GPX4 expression during MIRI. Given the critical role of GPX4 in ferroptosis (Yan et al., 2025), we further assessed the iron content, lipid peroxidation levels, and ferroptosis-related signaling pathways. MIRI triggered ferroptosis, as indicated by the increased tissue iron and LPO levels, along with elevated 12-HETE, 15-HETE, and 4-HNE levels (Figure 5C). Additionally, we observed a decreased expression of SLC7A11 and FSP1, coupled with the increased expression of acyl-coenzyme A (CoA) synthetase long-chain family member 4 (ACSL4,) TRF1, and DMT1 (Figures 5D, E). Notably, both GI and GSK2795039 effectively suppressed ferroptosis by reversing these changes (Figures 5C–E). In contrast, co-treatment with TBCA abolished the protective effects of GI against ferroptosis (Figures 5C–E). These findings suggest that GI mitigates MIRI-induced PANoptosis and ferroptosis by modulating NOX2 activity.

### 3.5 GI alleviated hypoxia/reoxygenation injury-induced cell injury and oxidative stress in primary cardiomyocytes

**FIGURE 6:**
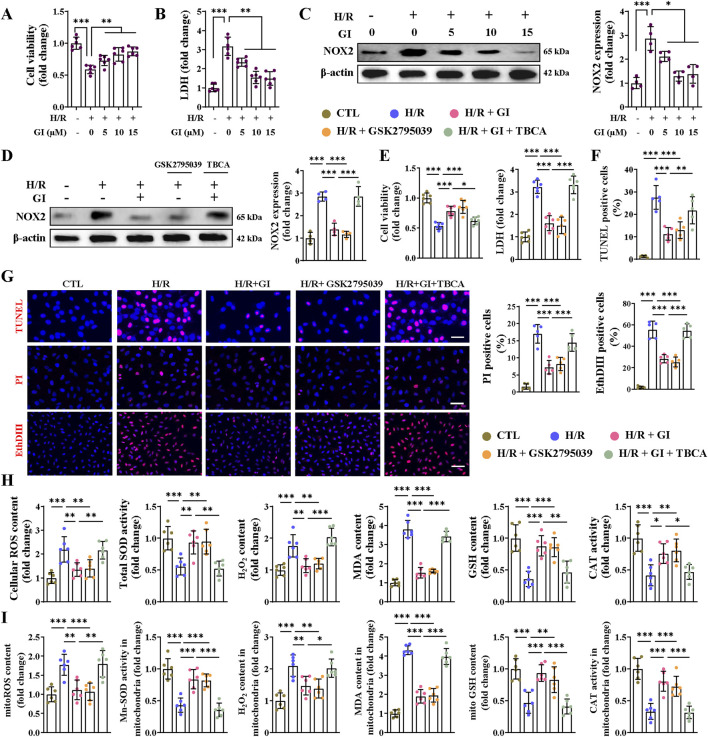
Effects of NOX2 on H/R injury-induced cell death and oxidative stress in cardiomyocytes. **(A)** The CCK8 assay was used for testing cell viability under the treatment of GI (0 μM, 5 μM, 10 μM, and 15 μM). **(B)** Statistical analysis of LDH levels. **(C)** Western blots of NOX2 expression *in vitro* under the influence of GI (0 μM, 5 μM, 10 μM, and 15 μM). **(D)** Western blots analysis of NOX2 expression *in vitro* under the treatment of GSK2795039 and TBCA. **(E)** Statistical analysis of cell viability and LDH levels. **(F,G)** Representative fluorescence images of TUNEL, PI, and Eth-III staining and statistical analysis of positive cells. **(H)** Effects of NOX2 activation on cellular contents of ROS, H_2_O_2_, GSH, and MDA and activities of CAT and SOD *in vitro*. **(I)** Effects of NOX2 activation on mitochondrial contents of ROS, H_2_O_2_, GSH, and MDA and activities of CAT and Mn-SOD *in vitro*. Four to six biological replicates were included in the experiment. Data are expressed as the mean ± standard error (SEM). One-way ANOVA test was applied for multi-group comparisons. * (p < 0.05), ** (p < 0.01), and *** (p < 0.001). Lactate dehydrogenase (LDH), tetrabromocinnamic acid (TBCA), reactive oxygen species (ROS), glutathione (GSH), malondialdehyde (MDA), catalase (CAT), superoxide dismutase (SOD), and manganese superoxide dismutase (Mn-SOD).

We attempted to further confirm that the protective effect of GI against MIRI was exerted on primary NMVCs with >95% purity (Supplementary Figure S1A). Hypoxia/reoxygenation (H/R) challenge markedly decreased cell viability with concomitant increases in LDH release and NOX2 expression (Figures 6A–C). GI pretreatment dose-dependently improved these injuries, with maximal protection at 10 μM (Figures 6A–C). This optimal concentration (10 μM) of GI was used in subsequent experiments. We then subjected cells to treatment with GSK2795039 and TBCA. GSK2795039 treatment recapitulated GI’s effects by reducing NOX2 expression, NOX activity, and LDH levels while improving cell viability (Figure 6D, E; Supplementary Figure S1B). Conversely, TBCA co-treatment completely abolished GI-mediated protection (Figure 6D, E; Supplementary Figure S1B). TUNEL, PI, and EthD-III staining confirmed that both GI and GSK2795039 reduced H/R injury-induced cell death, whereas TBCA eliminated GI’s benefits (Figure 6G). H/R injury promoted oxidative damage, which was manifested by elevated ROS, H_2_O_2_, and MDA levels alongside decreased SOD, GSH, and CAT activities at cellular and mitochondrial levels (Figures 6H, I). GI and GSK2795039 significantly attenuated these oxidative damages, while TBCA co-treatment reversed GI’s protective effects (Figures 6H, I).

### 3.6 GI alleviated H/R injury-induced oxidative stress by mediating the AMPK–PGC-1α–Sirt3 signaling pathway *in vitro*

**FIGURE 7:**
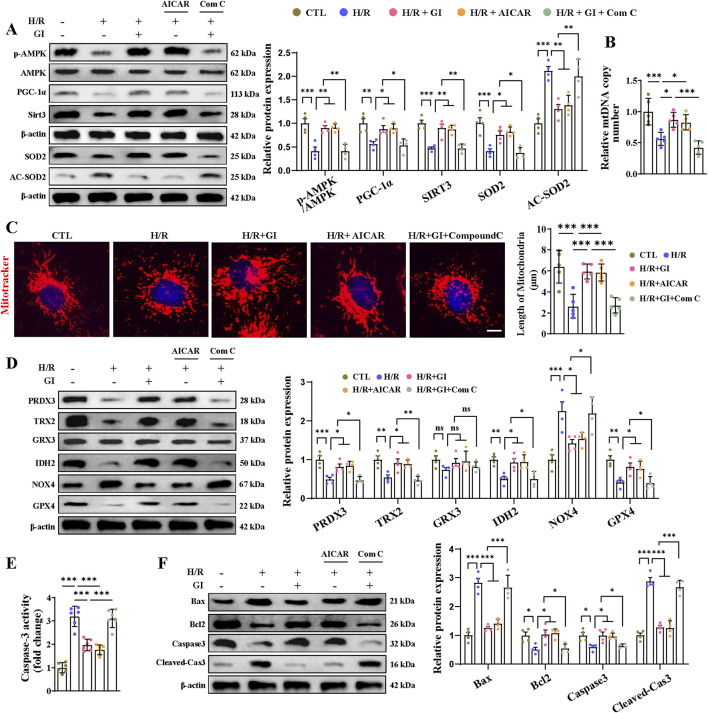
Effects of AMPK activation on H/R injury-induced oxidative stress and apoptosis in cardiomyocytes. **(A)** Western blot analyses for detecting the AMPK–PGC-1α–Sirt3 signaling pathway and the expression of SOD2 and Ac-SOD2. **(B)** Effects of AMPK activation on mtDNA copy number. **(C)** Representative image of MitoTracker staining. Scale bars = 5 μm. **(D)** Effects of AMPK activation on mitochondrial antioxidant/pro-oxidant-related proteins were examined by Western blotting. **(E)** Statistical analysis of caspase-3 activity. **(F)** Western blotting of the effects of AMPK activation on caspase-3, cleaved-caspase 3, Bax, and Bcl-2. Four to six biological replicates were included in the experiment. Data are expressed as the mean ± standard error (SEM). One-way ANOVA test was applied for multi-group comparisons. * (p < 0.05), ** (p < 0.01), and *** (p < 0.001).

Having demonstrated that GI activates the AMPK–PGC-1α–Sirt3 signaling pathway, we next investigated whether AMPK serves as the key downstream mediator of GI’s effects using the AMPK activator (AICAR) and inhibitor (Compound C). We found that AICAR reproduced GI’s protective effects, as shown by the enhanced AMPK–PGC-1α–Sirt3 pathway, increased mtDNA content, and improved mitochondrial morphology (Figures 7A–C). Meanwhile, co-treatment with Compound C largely abolished the benefits of GI (Figures 7A–C). In addition, AICAR decreased the level of NOX4 and elevated levels of PRDX3, TRX2, IDH2, and GPX4, similar to the effects of GI pretreatment (Figure 7D). In contrast, the inhibition of AMPK with Compound C abolished the protective effects of GI (Figure 7D). Taken together, all these results indicated that GI could protect primary cardiomyocytes against H/R injury-induced oxidative damage, largely dependent on AMPK activity.

### 3.7 Effect of GI on programmed cell death and AMPK downregulation in H/R-treated primary cardiomyocytes

**FIGURE 8:**
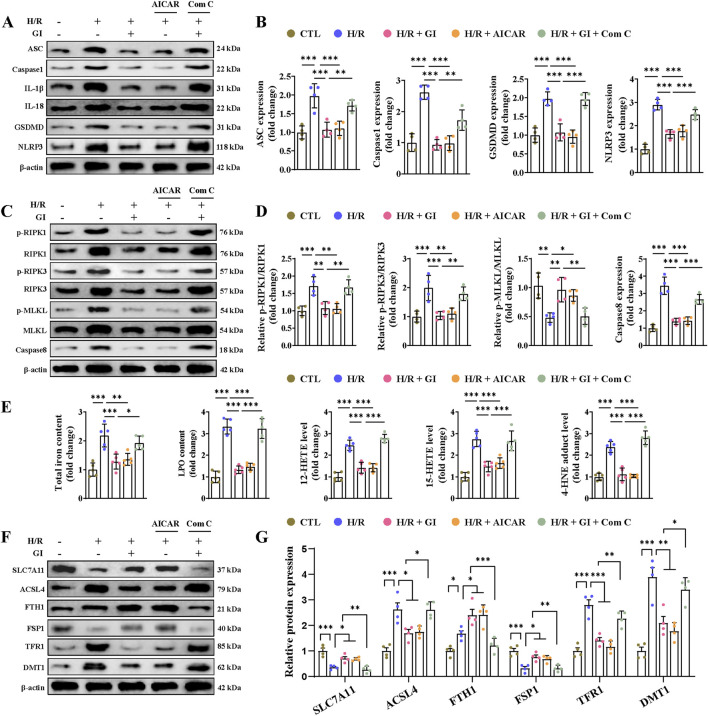
Effects of AMPK activation on H/R injury-induced pyroptosis, necroptosis, and ferroptosis in cardiomyocytes. **(A, B)** Western blot analyses of the effects of AMPK activation on the expression of NLRP3, ASC, caspase-1, GSDMD, IL-1β, and IL-18. **(C, D)** Western blot analyses of relative p-RIPK1, p-RIPK3, p-MLKL, and caspase levels under the influence of AMPK activation. **(E)** Contents of total iron and LPO and the levels of 12-HETE, 15-HETE, and 4-HNE in heart tissues. **(F, G)** Effects of AMPK activation on ferroptosis-related protein. Four to six biological replicates were included in the experiment. Data are expressed as the mean ± standard error (SEM). One-way ANOVA test was applied for multi-group comparisons. * (p < 0.05), ** (p < 0.01), and *** (p < 0.001).

Primary cardiomyocytes subjected to H/R injury also manifested PANoptosis and ferroptosis in accordance with the *in vivo* results. As for apoptosis, we found elevated levels of caspase-3 activity, increased expression of cleaved-caspase-3 and Bax, and lowered levels of Bcl-2 (Figures 7E, F). We also found elevated expression of IL-1β, IL-18 levels, caspase-1, ASC, NLRP3, and GSDMD, indicating the occurrence of pyroptosis (Figures 8A, B). Furthermore, confirmation of necroptosis was evidenced by a higher phosphorylation ratio of RIPK1, RIPK3, and MLKL and the higher expression of caspase-8 (Figures 8C, D). In addition, total iron and LPO content were both elevated after H/R injury, along with lowered SLC7A11 and FSP1 expression and higher ACSL4, TRF1, and DMT1 expression (Figures 8E–G). Pretreatment with GI or AICAR remarkably prevented H/R injury-induced PANoptosis and ferroptosis (Figures 7E, F). However, co-treatment with Compound C significantly inhibited the protective effects of GI on PANoptosis and ferroptosis (Figures 7E, F). These findings suggest that GI mitigates PANoptosis and ferroptosis in cardiomyocytes by modulating AMPK activation.

## 4 Discussion

Programmed cardiomyocyte death is a major pathological feature of reperfusion injury, and maintaining cell viability has become a critical therapeutic target in MIRI. Our study demonstrated that GI pretreatment significantly attenuated cardiomyocyte death, including PANoptosis and ferroptosis, in MIRI by mitigating mitochondrial oxidative stress and restoring impaired mitochondrial biogenesis. These cardioprotective effects were mediated through NOX2 suppression and activation of the AMPK–PGC-1α–Sirt3 signaling pathway. Specifically, GI pretreatment inhibited NOX2-derived oxidative damage, preserved AMPK–PGC-1α–Sirt3-dependent mitochondrial biogenesis, and consequently suppressed both PANoptosis and ferroptosis (Graphic Abstract).

During MIRI, cardiomyocytes undergo multiple forms of programmed cell death, a phenomenon that has been extensively studied. However, the signaling pathways governing these cell death processes often overlap or share common molecular components. In addition, targeting any single cell-death pathway may remain insufficient to fully mitigate cardiomyocyte death (Del Re et al., 2019). PANoptosis is a recently proposed programmed cell death modality characterized by the concurrent activation and mechanistic interplay of pyroptosis, apoptosis, and necroptosis (Gao et al., 2024). Emerging studies targeting PANoptosis inhibition have demonstrated promising therapeutic outcomes in renal, pulmonary, and cerebral ischemia–reperfusion injury models (Yan et al., 2023; Zhuang et al., 2025; He et al., 2023). In addition, targeting PANoptosis was also reported to be effective in alleviating doxorubicin-induced cardiotoxicity (Bi et al., 2022a). Ferroptosis was also a crucial form of programmed cell death and was identified in a variety of cardiac pathological conditions, including diabetic cardiomyopathy, myocardial infarction, and MIRI (Cai et al., 2023; Chen Z. et al., 2024; Chen Y. et al., 2024; Chen et al., 2023). This study demonstrates that PANoptosis and ferroptosis occur in cardiomyocytes during MIRI. Furthermore, GI exerts a cardioprotective effect by concurrently suppressing ferroptosis, pyroptosis, apoptosis, and necroptosis. The cardioprotective mechanism of GI may involve attenuating oxidative stress injury and preserving mitochondrial function. By modulating these processes, GI exerts downstream regulatory effects that concurrently inhibit multiple cell-death pathways (ferroptosis, pyroptosis, apoptosis, and necroptosis), thereby enhancing cardiomyocyte survival during MIRI.

It is well-established that reperfusion triggers an abrupt surge of ROS in cardiac tissue, leading to secondary cardiomyocyte death and exacerbation of the infarction zone (Li et al., 2021). As a critical organelle occupying ∼40% of the myocardial cytosolic volume, the mitochondrion is highly susceptible to reperfusion injury, which, in turn, triggers oxidative stress and mitochondria-dependent cell death (Gumpper-Fedus et al., 2022). Therefore, mitochondrial biosynthesis, a strategy for mitochondrial repair, is of great importance for cardiomyocyte protection. PGC-1α is a master regulator of mitochondrial biogenesis and an attractive therapeutic target (Fontecha-Barriuso et al., 2020). In addition, AMPK was identified as a key regulator in mitochondrial biogenesis during MIRI (Cai et al., 2022). Additionally, Sirt3, an NAD^+^-dependent mitochondrial deacetylase localized in the mitochondrial matrix, forms a positive feedback loop with AMPK to enhance mitochondrial biogenesis (Dikalova et al., 2020; Gao et al., 2020). This study demonstrated that GI pretreatment preserved mitochondrial biogenesis, thereby shielding cardiomyocytes from oxidative stress and attenuating PANoptosis and ferroptosis. Furthermore, our findings establish the AMPK–PGC-1α–Sirt3 pathway as a downstream signaling pathway of GI-mediated cardioprotection in MIRI, operating—at least in part—through the restoration of mitochondrial biogenesis and redox homeostasis.

Although mitochondrial dysfunction and programmed cell death in MIRI have been extensively studied, further investigation remains crucial given the persistent limitations in clinical treatment outcomes (Lesnefsky et al., 2017). This study demonstrates that GI mitigates oxidative stress injury by targeting NOX2, a key isoform of the NOX family known to regulate oxidative damage in various forms of cardiomyopathy (Lassègue et al., 2012). As an NADPH oxidase, NOX2 primarily generates ROS and regulates redox homeostasis. Notably, prior studies have established its detrimental effects in MIRI. Studies have demonstrated that NOX2-deficient mice exhibit significantly reduced infarction size compared to wild-type mice following MIRI (Braunersreuther et al., 2013). Critically, the crosstalk between NOX2 and AMPK signaling pathways has been shown to regulate apoptosis in MIRI (Qin et al., 2023). In our research, we further demonstrated that NOX2 inhibition was a potential mechanism by which GI attenuates oxidative stress injury and promotes mitochondrial biogenesis. More importantly, we found that, in addition to apoptosis, NOX2 promoted pyroptosis, necroptosis, and ferroptosis in MIRI. This also represents a key mechanism through which GI reduces cardiomyocyte death and myocardial infarction size via NOX2 suppression.

Our study also has several limitations. Although programmed cell death events in MIRI consistently occur within the first week, the exact timing of specific cell death types and the dominant death pattern at each time point remain under investigation. Similarly, the precise time window during which GI begins to exert its effects on particular death patterns remains unclear. Moreover, only pretreatment of GI is tested, but post-ischemia treatment would be more clinically relevant. Although this study demonstrates the protective effects of GI during acute MIRI, long-term functional outcomes (e.g., echocardiographic parameters and myocardial pathological remodeling) remain to be evaluated in future investigations. In addition, future studies should explore the roles of other NOX isoforms (e.g., NOX4) beyond NOX2 in MIRI. Finally, the results and conclusions were not tested in non-cardiomyocyte cells, such as fibroblasts, endothelial cells, or immune cells, which also contribute to MIRI pathology.

Collectively, GI attenuated PANoptosis and ferroptosis during MIRI by eliminating ROS burst and preserving mitochondrial biogenesis. NOX2 acts as the key effector of GI, while the AMPK–PGC1α–Sirt3 axis mediates its downstream protective signaling. These findings identify GI as a promising therapeutic strategy for mitigating programmed cell death in MIRI.

## Data Availability

The datasets presented in this study can be found in online repositories. The names of the repository/repositories and accession number(s) can be found in the article/Supplementary Material.

## Abbreviations

AMPK, AMP-activated protein kinase; AICAR, 5-aminoimidazole-4-carboxamide ribonucleotide; Ac-SOD, acylated superoxide dismutase; ACSL4, acyl-coenzyme A (CoA) synthetase long-chain family member 4; CAT, catalase; GSH, glutathione; GPX4, glutathione peroxidase 4; GSSG, glutathione disulfide; GRX3, glutaredoxin-3; GI, gypensapogenin I; GSDMD, gasdermin D; IDH2, isocitrate dehydrogenase 2; LVFS, left ventricular fractional shortening; LVEF, left ventricular ejection fraction; MDA, malondialdehyde; MIRI, myocardial ischemia–reperfusion injury; MLKL, mixed lineage kinase domain like; NOX2/4, NADPH oxidase 2/4; NPSH, non-protein thiol; NMVCs, neonatal mouse ventricular cardiomyocytes; PRDX3, peroxiredoxin 3; ROS, reactive oxygen species; RIPK1/3, receptor-interacting serine-threonine protein kinases 1/3; SH, total thiol; TBCA, tetrabromocinnamic acid; TRX2, thioredoxin 2; TRXR, thioredoxin reductase.
