# Supplementary figures and images for: Gypensapogenin I alleviates PANoptosis, ferroptosis, and oxidative stress in myocardial ischemic–reperfusion injury by targeting the NOX2/AMPK pathway
Source: Front Cell Dev Biol. 2025 Jul 22;13:1623846. doi: 10.3389/fcell.2025.1623846 (PMC12321891; doi:10.3389/fcell.2025.1623846)

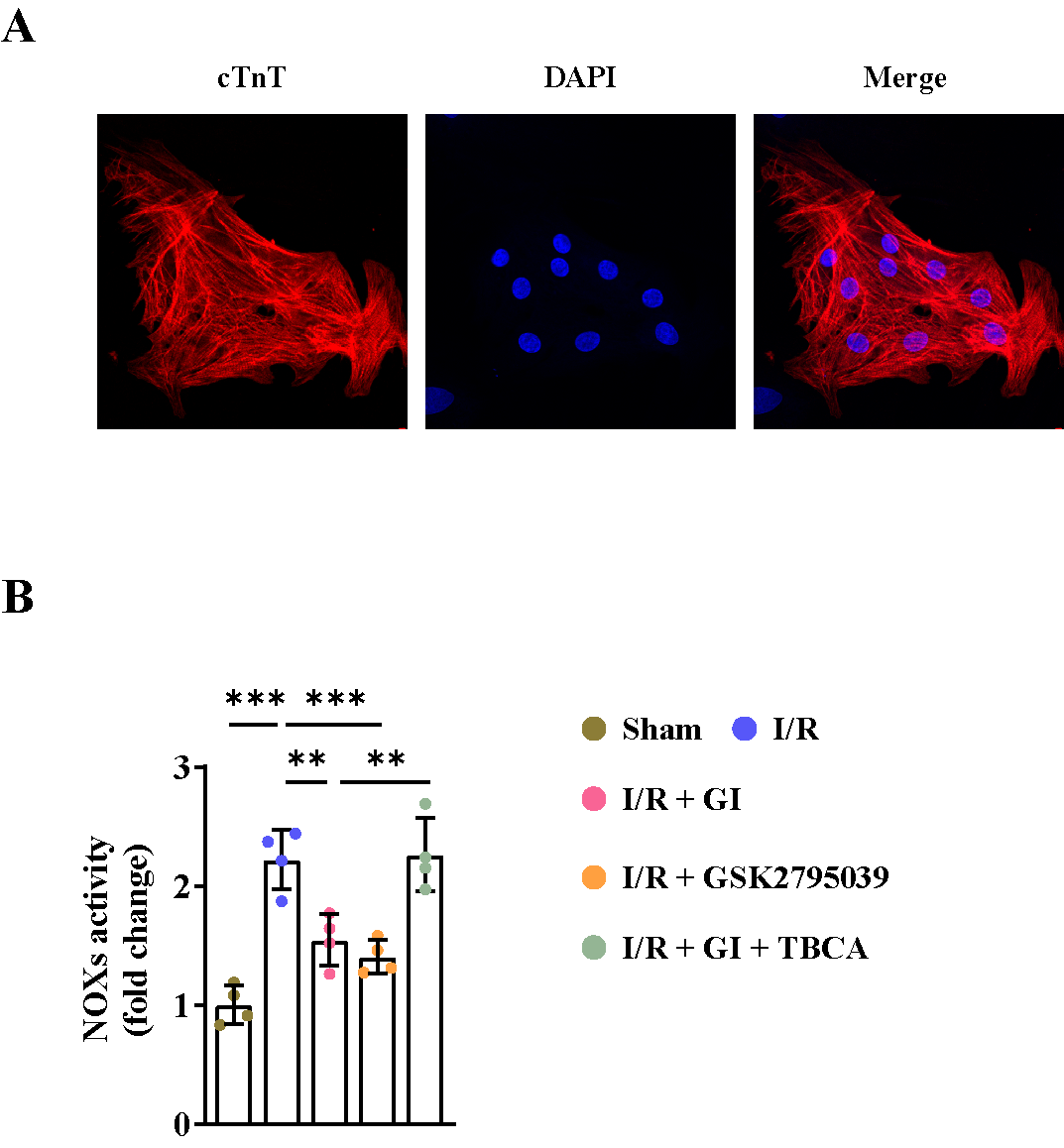

Supplement: Supplementary file 1 [file Image1.tif]
